# Aggressive fibromatosis response to tamoxifen: lack of correlation between MRI and symptomatic response

**DOI:** 10.1186/s13569-018-0100-3

**Published:** 2018-05-14

**Authors:** M. Libertini, I. Mitra, W. T. A. van der Graaf, A. B. Miah, I. Judson, R. L. Jones, K. Thomas, E. Moskovic, Z. Szucs, C. Benson, C. Messiou

**Affiliations:** 1 Sarcoma Unit, Royal Marsden NHS Foundation Trust, London, UK; 2 Department of Radiology, The Royal Marsden NHS Foundation Trust, 203 Fulham Road, London, SW3 6JJ UK; 3 Institute of Cancer Research, Sutton, UK

**Keywords:** Aggressive fibromatosis, Anti-oestrogen therapy, Symptoms, MRI features, T2 weighted signal

## Abstract

**Background:**

One of the commonly used systemic agents for the treatment of aggressive fibromatosis is the anti-oestrogen drug tamoxifen. However, data on efficacy and optimum methods of response assessment are limited, consisting mainly of small case series and reports.

**Methods:**

A retrospective database was used to identify consecutive patients diagnosed with aggressive fibromatosis (AF) and treated with tamoxifen plus/minus non-steroidal anti-inflammatory drugs at our tertiary referral centre between 2007 and 2014. MRI and symptom changes were recorded.

**Results:**

Thirty-two patients (13 male 19 female, median age 41 years) were included. Median duration of treatment with tamoxifen was 316 days. Of 9 patients with progressive disease by RECIST 1.1 (28%): 4 patients experienced worsening symptoms; 3 patients had improved symptoms and 2 had no change in symptoms. Of 22 patients with stable disease (69%): 11 had no change in symptoms; 6 had improved symptoms and 5 patients had worsening symptoms. One patient achieved a partial response with improved symptoms.

**Conclusions:**

No relationship was identified between symptomatic benefit and response by RECIST 1.1 on MRI. Prospective studies in AF should incorporate endpoints focusing on patient symptoms.

## Background

Aggressive fibromatosis (AF), also named desmoid-type fibromatosis, is characterised by monoclonal myofibroblastic proliferation in soft tissues. It is a rare disease accounting for 3% of all soft tissue neoplasms, with an incidence of 2–5 people per million per year [1]. It has a female predominance and a peak incidence in the third to fourth decades [2]. AF is often sporadic, however, there is a reported increased incidence of 3.5–32% in patients with familial adenomatous polyposis (FAP) or Gardner’s variant [3, 4]. AF is usually solitary but multifocal tumours have been reported [5]. It may arise from any anatomical site, commonly the extremities, abdominal and chest wall and paravertebral tissues [6, 7]. Although AF is slow growing without metastatic potential, its unpredictable behaviour, propensity for progressive infiltration and local invasion makes treatment challenging. Currently there is no established evidence-based approach to treatment [8], although a consensus approach based on wide consultation with physicians and patient groups has been published [9]. Active surveillance is now often used in asymptomatic cases [10]. High local recurrence rates of 15–50% [11–14] up to 87% [15] in younger patients, despite apparently complete resection, have reduced the popularity of surgical resection as initial management. Radiotherapy can help improve local control [16] however, side effects, including radiation-induced malignancies have to be considered especially in young patients [17].

Drugs used in the treatment of AF, include hormonal therapy (e.g. tamoxifen and toremifene) [18], Non steroidal antinflammatory drugs (NSAIDs) and cytotoxic chemotherapeutic agents, such as anthracycline-based regimens [19] (including pegylated liposomal doxorubicin [20]) and vinblastine plus methotrexate [21]. Tyrosine kinase inhibitors, including imatinib [22], sorafenib [23] and pazopanib [24] can also play a role in the treatment of AF. A recent phase I study demonstrated demonstrated promising efficacy of a γ-secretase inhibitor in desmoid tumours [25].

For many centres, first line systemic treatment of non-resectable or symptomatic desmoid-type fibromatosis consists of hormonal manipulation, with or without a NSAID. Particularly in centres within the United Kingdom, this is heavily influenced by lack of reimbursed alternatives, availability and the low side effect profile. Immunohistochemical studies have demonstrated the presence of oestrogen receptor-beta in 90% of desmoid-type tumours [26]. This is supported by the tendency of fibromatosis to occur more often in women, particularly during pregnancy/within 1 year post partum [27], or on oral contraception, and there are reports of spontaneous regression during menopause and post-partum [2, 28]. Several publications have documented the effectiveness of hormonal manipulation in AF treatment [29–32]. Despite the lack of randomised prospective data, it has been reported that antioestrogen therapy can be effective in about half of patients [18]. Tamoxifen is a non-steroidal triphenylethylene derivative that binds to oestrogen receptors. One suggested mechanism for the anti-proliferative action of tamoxifen is regulation of the synthesis of the cytokine transforming growth factor-β (TGF-β) [32] and its receptors, which are also involved in AF pathogenesis.

There are several means of monitoring treatment response, including clinical evaluation of tumour size and symptoms as well as radiological. The Response Evaluation Criteria in Solid Tumours (RECIST 1.1) [33] are currently employed within clinical trials. Magnetic resonance imaging (MRI) has become the imaging modality of choice for soft tissue lesions, due to better evaluation of the tumour and its relationship with surrounding structures such as nerves and vessels. Given the chronic nature of the disease, the lack of radiation exposure makes MRI ideal for follow up studies. Furthermore, in lesions undergoing radiation or drug therapy, MR surveillance has been used to assess response to treatment with a decrease in T2-weighted signal and lesion size being suggested as indicators of treatment response [34].

The variable content of spindle cells, collagen and myxoid tissue of AF correlates with the observation that these lesions often show heterogeneous signals on MRI [35]. In particular, the highly cellular, actively growing lesions tend to be of high signal on T2-weighted MR images [6, 36, 37]. Interspersed low signal bands correspond with the collagen bundles. As the lesion matures, the increase in collagen deposition and decreased cellularity result in a decrease in T2 signal [38, 39]. However to date it has not been possible to predict behaviour based on MRI signal [40, 41].

The main aims of this study were to assess MRI response and symptom control in patients with AF treated with tamoxifen with or without NSAIDs.

## Methods

### Patient selection

The prospectively collected Royal Marsden Hospital sarcoma database was used to identify consecutive patients diagnosed with AF and treated with tamoxifen at our tertiary referral centre between 2007 and 2014. Institutional approval was obtained. Inclusion criteria were patients aged 18 years and over, treated with tamoxifen, with a baseline and at least one follow-up MRI scan. Demographic data, disease characteristics, previous treatments, date of starting and stopping tamoxifen, toxicity and clinical symptoms were collected from clinical notes. Descriptive statistical analysis was applied: progression-free survival (PFS) was calculated using the Kaplan–Meier method.

### Imaging data

Baseline MRI images, defined as the last MRI performed prior to tamoxifen treatment, and subsequent follow up MRI images were identified for each patient. Where available a pre-baseline MRI was also collected. On average 3 (range 2–6) follow up MRIs were assessed for each patient. The minimum MRI protocol for inclusion in the study was axial T1W, T2W, STIR and coronal T2W and STIR images. All images were re-reviewed by a specialist soft tissue radiologist (CM). Tumour size, RECIST 1.1 assessment and T2-weighted signal changes were documented at each time point.

## Results

Between 2007 and 2014 a total of 35 patients were treated with tamoxifen at the Royal Marsden Hospital. Baseline imaging was not available for 3 patients, and they were therefore not eligible for this study. Of the remaining 32 cases, the median age at the time of commencing tamoxifen was 41 years (range 19–68 years). There was a 3:2 female to male ratio [19 (60%): 13 (40%)]. One patient (3%) had a diagnosis of FAP. The most common site of origin was limb and limb girdle (18; 56%), followed by chest wall (5; 15%), pelvis (3; 10%), abdominal wall (3; 10%), paravertebral tissues (1; 3%) and head/neck (2; 6%).

Fourteen patients (44%) received tamoxifen as first-line treatment. Eighteen of 32 patients (56%) had been treated previously, with surgery, radiotherapy, steroid injections, NSAIDs or doxorubicin chemotherapy. Patient and disease characteristics are summarised in Table 1.

**Table 1:** Patient and disease characteristics

	#	(%)	[range]
Number of patients	32		
Male	14	(40%)	
Female	19	(60%)	
Median age on starting tamoxifen	41		[19–68]
Site of origin			
Extremities (including girdle)	18	(56%)	
Chest wall	5	(15%)	
Pelvis	3	(10%)	
Abdominal wall	3	(10%)	
Paravertebral	1	(3%)	
Head and neck	2	(6%)	
Sporadic disease	31	(97%)	
FAP-associated	1	(3%)	
Previous treatments	18	(56%)	
Local treatment			
Surgery	10	(31%)	
Surgery and RT	7	(21%)	
RT alone	0		
Systemic treatment	4	(12%)	
No previous treatment	14	(44%)	

Tamoxifen treatment was started due to tumour growth and worsening symptoms, mostly characterised by pain, including neuropathic and somatic pain, and decreased range of movement. Four patients (12%) were asymptomatic at the time of starting tamoxifen.

The average length of time on tamoxifen was 316 days, ranging from 1 month to 3 years (33–997 days). Tamoxifen dosages used were 40 mg (15 patients), 20 mg (15 patients), 10 mg (1 patient) and unknown (1 patient). Twenty-four patients (75%) received tamoxifen in association with a NSAID (naproxen or diclofenac). Thirteen of the 32 patients (41%) suffered from tamoxifen-related side effects, most commonly hot flushes and mood swings. Two patients had their 40 mg dose reduced to 20 mg due to side effects. One of those was a 40-year old male who experienced increased tiredness and the other a 27-year old male who suffered from mood swings, fatigue and hot flushes.

The most common reasons for stopping tamoxifen were tumour progression (10, 31%), and grade 2–3 side effects (8, 25%), such as hot flushes, mood swings and fatigue. Four patients (13%) stopped due to lack of any perceived benefit and 3 (9%) due to worsening symptoms. One patient with stable disease stopped tamoxifen as she was planning on starting a family and she was aware of possible associated birth defects [42]. One patient stopped tamoxifen because of pregnancy. Two patients died for reasons unrelated to AF. One patient stopped tamoxifen due to side effects, and subsequently received an anti-tumour necrosis factor (anti-TNF)-α agent, adalimumab [43], for his rheumatoid arthritis and the AF decreased in size. Two patients continue on tamoxifen.

**Fig. 1:**
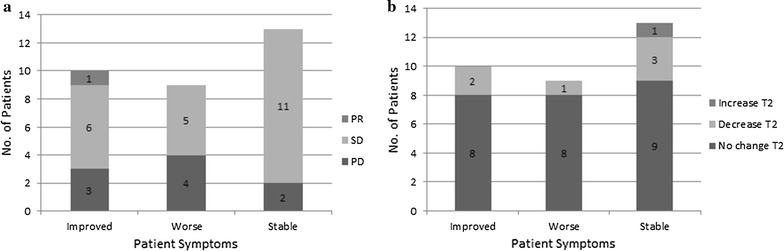
Barcharts of patient symptoms and corresponding RECIST 1.1 status (**a**) and T2W MRI signal changes (**b**)

Tamoxifen with or without a NSAID, resulted in symptom improvement in 10 patients (31%)—5 patients on 40 mg tamoxifen, 4 patients on 20 mg tamoxifen and 1 patient on 10 mg tamoxifen (8 of these 10 patients were also taking NSAIDs). Worsening symptoms were experienced by 9 patients (28%)—2 patients on 40 mg tamoxifen, 6 patients on 20 mg tamoxifen and 1 unknown (6 of these 9 patients were also taking NSAIDs). Thirteen patients did not experience any change in symptoms (41%)—9 patients on 40 mg tamoxifen, 4 patients on 20 mg tamoxifen (10 of these 13 patients were also taking NSAIDs). The majority of patients with symptomatic benefit did not have significant changes in size or signal on MRI (Fig. 1a, b).

**Fig. 2:**
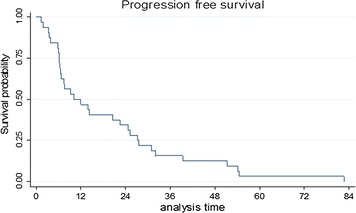
Kaplan–Meier progression free survival analysis after treatment with tamoxifen. Median PFS was 10 months (95% CI 6.4–24.6)

The median tumour size on starting tamoxifen was 60 mm (range 23–165 mm). There was a varied response in tumour size. Eighteen patients (56%) had increase in tumour size, 6 patients (19%) had a reduction in tumour size and 8 patients (25%) had no change in tumour size. By RECIST 1.1, 9 patients (28%) had progressive disease—6 patients on 40 mg tamoxifen, 2 patients on 20 mg tamoxifen and 1 unknown; 22 patients (69%) had stable disease—8 patients on 40 mg tamoxifen, 13 patients on 20 mg tamoxifen, 1 patient on 10 mg tamoxifen and 1 patient (3%) had a partial response—40 mg tamoxifen. Median progression-free survival (PFS) per RECIST 1.1 was 10 months with (95% CI 6.4–24.6); PFS at 1 and 2 years was 50% (95% CI 32–66) and 34% (95% CI 19–51), respectively (Fig. 2), with a median follow-up of 45.5 months (range 14–105).

We divided our series into three RECIST 1.1 criteria-based groups: progressive disease, stable disease and partial response. Of 9 patients with progressive disease (28%): 4 patients experienced worsening symptoms with increased pain; 3 patients had an improvement in pain and increase in range of movement and no change in symptoms was observed in the remaining 2 patients. T2 signal increase was observed in 1 case, which correlated with clinical deterioration; in 8 of 9 cases there was no change in T2 signal. Of 22 patients with stable disease (69%): 11 had no change in symptoms with T2 signal reduction in 3 cases and increase in 1; 6 experienced symptom improvement: 1 of 6 had a correlating T2 signal reduction. Five patients complained of worsening symptoms without any change in T2 signal.

**Fig. 3:**
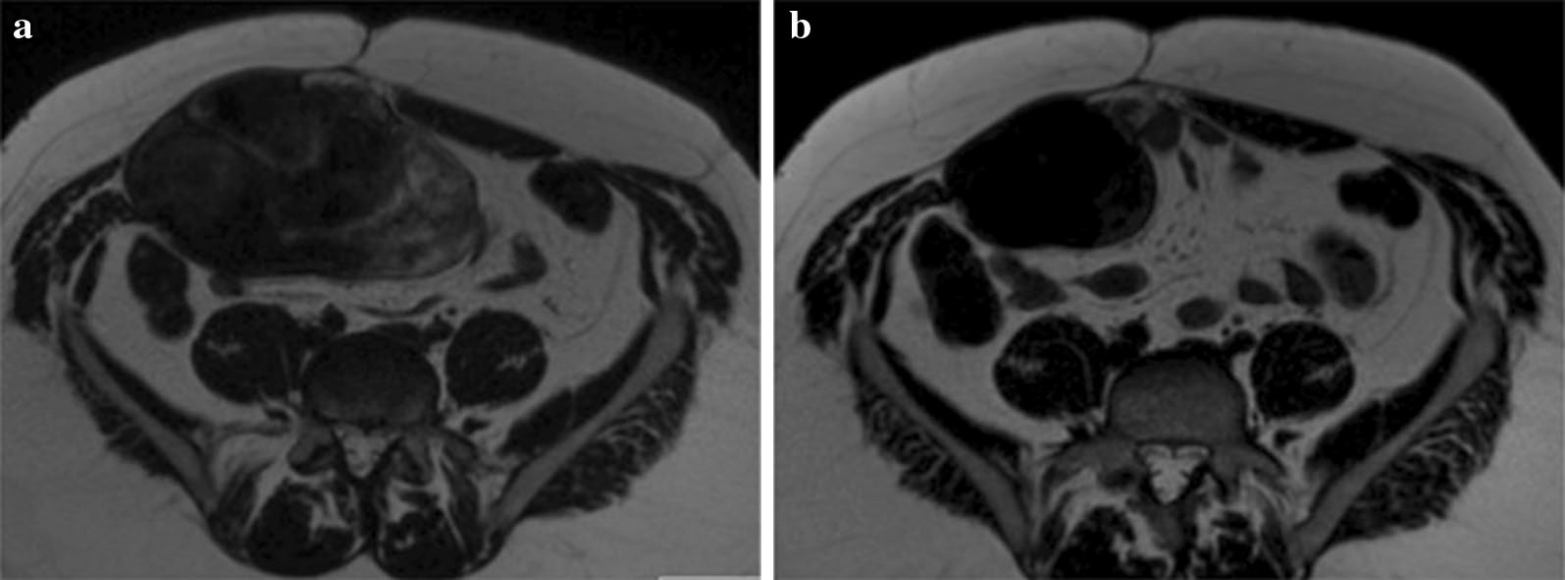
Axial T2 weighted MRI images showing right anterior abdominal wall fibromatosis in a 35-year old male (**a**) and the corresponding MRI after 7 months of treatment with tamoxifen (**b**) show decrease in size and T2 signal

One patient achieved a partial response (3%). This patient was a 35-year old male affected by AF involving the right anterior abdominal wall with abdominal pain at baseline. This patient is still on treatment, and has completed 476 days of therapy at the time of analysis. After 18 months of treatment, he experienced an improvement in symptoms with a reduction in pain. The symptomatic improvement corresponded with a decrease in size and T2 signal on MRI (Fig. 3).

Additional pre-baseline MRI scans were available for 14 patients. Thirteen out of 14 patients’ tumours were increasing in size prior to starting tamoxifen. Six continued to increase following tamoxifen; 5 showed some decrease in size; 2 became stable having demonstrated growth prior to starting tamoxifen.

## Discussion

Aggressive fibromatosis is a challenging disease with an unpredictable behaviour. The unsatisfactory outcomes of surgery and the fact that growth arrest and regression can occur spontaneously have led to the increased adoption of active surveillance as the initial approach to management [44]. However hormonal manipulation has been commonly used particularly in the United Kingdom as first-line systemic therapy in AF [10].

Our study of 32 patients is limited by the retrospective design and the collection of symptomatic changes by retrospective case note review rather than prospective dedicated questionnaires. However, to our knowledge, this is the largest single series evaluating the relationship between symptoms and MRI response in patients with AF treated with tamoxifen with/without NSAIDs.

Within this case series, 14 of the 32 patients (44%) received tamoxifen as first-line treatment. This could reflect the complexity of the cases referred to our tertiary centre but also the focus on preserving function and quality of life. Furthermore, we found that 8 of the 32 patients (25%) had to stop tamoxifen due to side effects, which is higher than the previously reported rate of 10% [45] and remarkable given the, for this condition, relatively low dose of tamoxifen prescribed in our patients [29, 46].

In our series 18 of the 32 patients (56%) had an increase in tumour size and among these, 28% were defined as disease progression by RECIST 1.1. This is in keeping with the rates of disease progression stated in a systematic review [18]. However, our stable disease and partial response rates of 69 and 3% respectively, do not correlate with the 18 and 58% rates previously quoted, raising the possibility of an overestimation in the reported efficacy of tamoxifen in AF. Importantly, 31% of patients did report symptomatic benefit, although the positive contribution of NSAIDs cannot be excluded. Seventy-five percent of cases in this series were treated with concomitant NSAIDs, which may have influenced the results, since prostaglandin blockage has been shown to provide some benefit in the treatment of AF [47].

Interestingly, one patient treated with a fully human monoclonal antibody tumor necrosis factor inhibitor (TNFi), adalimumab, used for rheumatoid arthritis showed a response after tamoxifen discontinuation, suggesting either a possible late tamoxifen effect or a role of immunomodulation in AF pathogenesis and treatment.

Among the population of patients with stable disease, representing the most heterogeneous group, we found a discordance between clinical symptoms and MRI T2 changes. This could be explained by the fact that cases with a slight increase/decrease in size are included within the category of RECIST 1.1 stable disease.

Although MRI is accepted to be the best imaging modality for visualising AF [38] this study suggests that it is less useful in demonstrating therapeutic benefit on tamoxifen ± NSAIDs. This study has not demonstrated a clear relationship between MRI features (size/signal) and reported symptoms. This limitation may not be restricted to assessment of tamoxifen effects as Sheth et al. also reported that RECIST were not sensitive to clinically determined response in 23 patients treated with a variety of local and systemic therapies [48]. Although there is thought provoking evidence that FDG PET/CT gives some early indication of response in patients treated with imatinib, the risk:benefit ratio of the radiation doses involved must be given careful consideration particularly where multiple assessments for non malignant pathology are performed, especially in young patients [49].

Our study suggests that symptoms are arguably the most important indicators of response to tamoxifen in patients with AF. According to this observation, the incorporation of prospective validated pain scores and functional assessment tools into the evaluation of treatment in this disease would give a better indication of therapeutic benefit. This is particularly critical for the design of prospective AF studies where we suggest that endpoints should focus on patient symptoms. However, some experience with MRI in patients treated on pazopanib indicate that MRI may be more useful in assessing response to other agents [25].

For those patients with previous pre-baseline images available for comparison, a few cases showed a decrease in the rate of tumour growth. However, it is difficult to extrapolate definitive conclusions from these data because the number of patients with pre-baseline images was low and AF is known to have such a varied natural history with prolonged periods of stabilisation and eventual regression in nearly 28% of cases in one reported series [50].

## Conclusions

We showed for the first time that symptomatic benefit, MRI T2 signal changes and tumour size correlate poorly. Therefore, MRI is of limited value in assessing therapeutic benefit in patients treated with tamoxifen ± NSAIDs. This highlights the importance of robust systems to collect data on patients’ symptoms and quality of life.
